# Open versus Endovascular Repair of Abdominal Aortic Aneurysm in the Elective and Emergent Setting in a Pooled Population of 37,781 Patients: A Systematic Review and Meta-Analysis

**DOI:** 10.1155/2014/149243

**Published:** 2014-04-02

**Authors:** Dustin M. Thomas, Edward A. Hulten, Shane T. Ellis, David M. F. Anderson, Nathan Anderson, Fiora McRae, Jamil A. Malik, Todd C. Villines, Ahmad M. Slim

**Affiliations:** ^1^Cardiology Service MCHE-MDC, Brooke Army Medical Center, 3551 Roger Brooke Drive, San Antonio, TX 78234-6200, USA; ^2^Cardiology Service, Walter Reed National Military Medical Center, Bethesda, MD 20889, USA; ^3^Medicine, USUHS, 3551 Roger Brooke Dr, San Antonio, TX 78234, USA

## Abstract

*Background*. We evaluated the incidence of mortality and myocardial infarction (MI) in endovascular repair (EVAR) as compared to open aneurysm repair (OAR) in both elective and ruptured abdominal aortic aneurysm (AAA ) setting. *Methods*. We analyzed the rates of 30-day mortality, 30-day MI, and hospital length of stay (LOS) based on comparative observation and randomized control trials involving EVAR and OAR. *Results*. 41 trials compared EVAR to OAR with a total pooled population of 37,781 patients. Analysis of elective and ruptured AAA repair favored EVAR with respect to 30-day mortality with a pooled odds ratio of 0.19 (95% CI 0.17–0.20; *I* ^2^ = 88.9%; *P* < 0.001). There were a total of 1,835 30-day MI events reported in the EVAR group as compared to 2,483 events in the OAR group. The pooled odds ratio for elective AAA was 0.74 (95% CI 0.58–0.96; *P* = 0.02) in favor of EVAR. The average LOS was reduced by 296.75 hrs (95% CI 156.68–436.82 hrs; *P* < 0.001) in the EVAR population. *Conclusions*. EVAR has lower rates of 30-day mortality, 30-day MI, and LOS in both elective and ruptured AAA repair.

## 1. Background

Abdominal aortic aneurysm (AAA) is a prevalent threat, affecting approximately 5% of males over the age of 65. Open aneurysm repair (OAR) has been performed since the 1950s with a 30-day mortality of 4–12% [1]. Since the early 1990s, endovascular repair (EVAR) of both elective and ruptured AAA has steadily increased. This was driven predominantly by early data reporting lower 30-day mortality rates of 1-2% [2]. Subsequent long-term follow-up data from both the EVAR 1 trial group as well as the DREAM trial suggested the mortality curves become equivalent as early as 1 year [3, 4]. Previous meta-analysis looked to address the 30-day mortality rates based on age and surgical experience as well as defining operative complications.

We performed a systemic review of the literature to analyze the rates of 30-day mortality, 30-day myocardial infarction, and hospital length of stay based on comparative observation and randomized control trials involving endovascular and open approach to elective and ruptured AAA repair.

## 2. Methods

### 2.1. Data Sources and Study Selection

**Figure 1:**
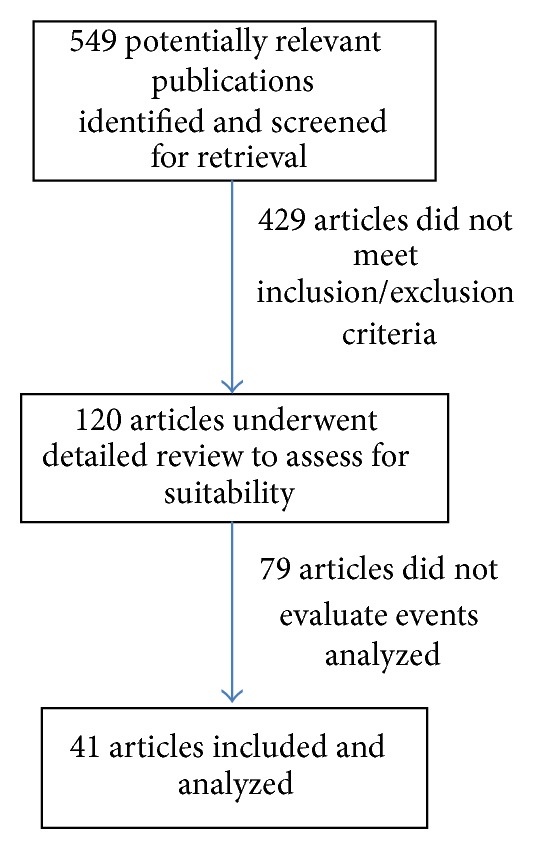
Flow diagram of articles evaluated that did not meet inclusion/exclusion criteria during the search period.

Two reviewers independently conducted the literature search and extraction of relevant articles from MEDLINE database, Embasse database, and Cochrane library for English language studies in humans older than 18 years of age. The date of the last search was obtained on July 1, 2012. We used the text words and related Medical Subject Headings terms: EVAR, outcome, mortality, morbidity, and endovascular aortic repair. We also searched relevant references cited in reviewed articles. We followed the guidelines of the Meta-Analysis of Observational Studies in Epidemiology Group in both the execution and the reporting of our findings [5]. Initial phase included review of the title and abstract of potentially relevant articles for inclusion in the review before retrieval of the full articles. We included observational studies and randomized control trials in adults which compared EVAR to OAR for repair of either ruptured or elective AAA (Figure 1). We included articles which reported rates of 30-day mortality, perioperative myocardial infarction rates, and hospital length of stay. We excluded duplicate publications or serial publications reporting on the same study population. Disagreements were resolved by consensus.

### 2.2. Data Extraction and Quality Assessment

The following characteristics of the study were extracted: author, year, design, sample size, elective or ruptured AAA, AAA diameter, ICU length of stay, hospital length of stay, operative time, estimated operative blood loss, perioperative transfusion requirement, and age. Outcomes abstracted included incidence of perioperative myocardial infarction (MI), acute kidney injury (AKI), colon ischemia, leg ischemia, operative conversion rates to open repair, rates of endograft leak (specifically type 1 and type 2 endoleaks), and 30-day mortality.

All statistics were performed using Stata (Version 11, College Station, Texas). Two reviewers independently abstracted data and disagreements were resolved by consensus. Two reviewers independently rated study quality using the Newcastle-Ottawa scale for the assessment of the quality of observational studies [6].

### 2.3. Data Synthesis

Our principal abstracted measure of effect was the odds ratio of a given outcome comparing EVAR to OAR. Univariate unadjusted outcomes were used and combined odds ratios were calculated to pool the odds ratio of individual study outcomes within the respective groups. Studies with no outcome event in either group were included for estimation of absolute incidence but could not be considered for calculation of the pooled odds ratio. Outcomes were pooled using a random effects (DerSimonian and Laird) model.

Heterogeneity was assessed by using the *I* ^2^ statistic. The *I* ^2^ statistic provides an estimate of the amount of variance due to heterogeneity rather than chance and is based on the traditional measure of variance, the Cochrane *Q* statistic. We conducted stratified analyses in order to assess potential confounders' contribution to heterogeneity, including age, gender, and study quality (≤ or > the median overall Newcastle-Ottawa score as well as individual component analysis). Publication bias was assessed using Begg and Egger's method. All *P* values were two-sided with an alpha of 0.05.

## 3. Results

**Figure 2:**
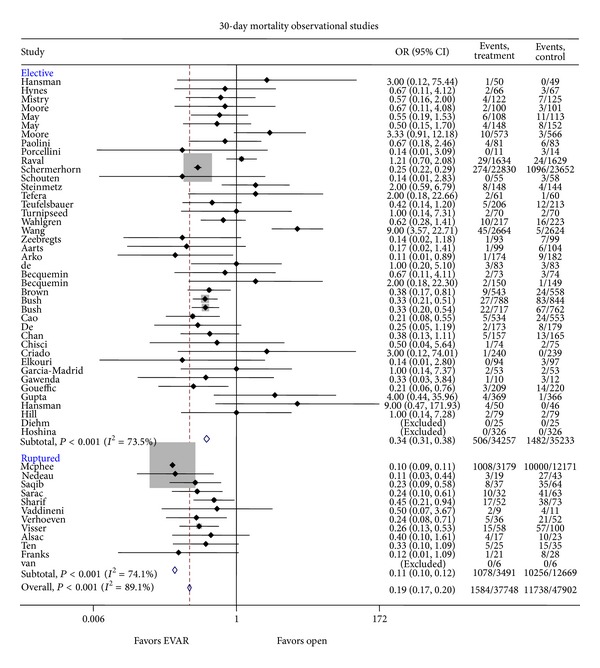
Odd ratio of mortality risk in OAR as compared to EVAR in the elective as well as the emergent (ruptured) setting.

**Figure 3:**
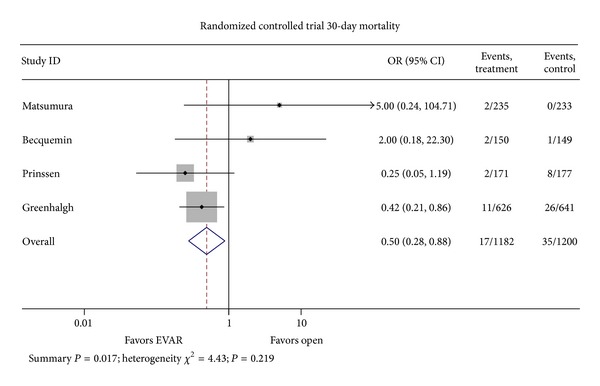
Odd ratio of mortality risk in OAR as compared to EVAR randomized clinical trials.

Baseline demographic data from the articles analyzed are included in Table 1 to include median patient age, AAA mean diameter, and study population size when reported. Forty-one trials (Figure 2) compared EVAR to OAR in elective AAA patients (7–46). A total of 1,594 deaths were reported in the EVAR population (506 deaths in the elective group) and mortality at 30 days favored EVAR with a pooled odds ratio of 0.34 (95% CI 0.31–0.38; *I* ^2^ = 73.5%; *P* < 0.001). Eleven trials (Figure 2) compared EVAR to OAR in ruptured AAA patients [7–18]. EVAR was superior to OAR in the ruptured AAA population as well with a pooled odds ratio of 0.11 (95% CI 0.10–0.12; *I* ^2^ = 74.1%; *P* < 0.001). Overall analysis of elective and ruptured AAA repair favored EVAR with respect to 30-day mortality with a pooled odds ratio of 0.19 (95% CI 0.17–0.20; *I* ^2^ = 88.9%; *P* < 0.001). Looking specifically at 30-day mortality rates in the 4 randomized controlled trials included in the elective analysis, EVAR was favored with a pooled odds ratio of 0.50 (95% CI 0.28–0.88; *P* = 0.017; *I* ^2^ = 4.43; *P* = 0.219) (Figure 3) [2, 19–21].

**Figure 4:**
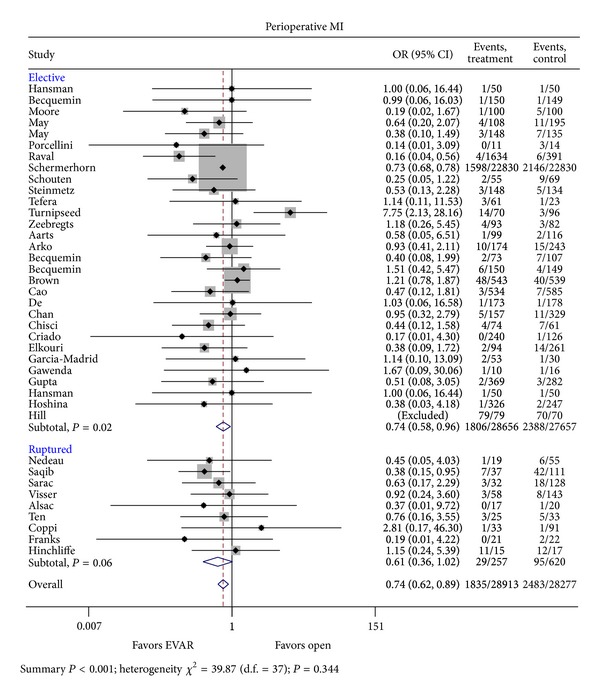
Odd ratio of myocardial infarction risk in OAR as compared to EVAR in the elective as well as the emergent (ruptured) setting.

Twenty-nine trials of elective AAA repair and 9 ruptured AAA repair trials were included in the analysis of MI (Figure 4) [20, 22–45]. There were a total of 1,835 events (1,806 events in the elective AAA repair population) reported in the EVAR group compared with 2,483 events (2,388 events in the elective AAA repair population) in the OAR group. The pooled odds ratio for elective AAA was 0.74 (95% CI 0.58–0.96; *P* = 0.02) in favor of EVAR. Ruptured AAA was 0.61 (95% CI 0.36–1.02; *P* = 0.06) suggesting a trend in favor of EVAR though did not rise to the level of statistical significance [8–10, 14–18, 46]. Pooled analysis of both elective and ruptured studies give an overall odds ratio of 0.74 (95% CI 0.62–0.89; *X* ^2^ = 39.87; *P* = 0.344).

**Figure 5:**
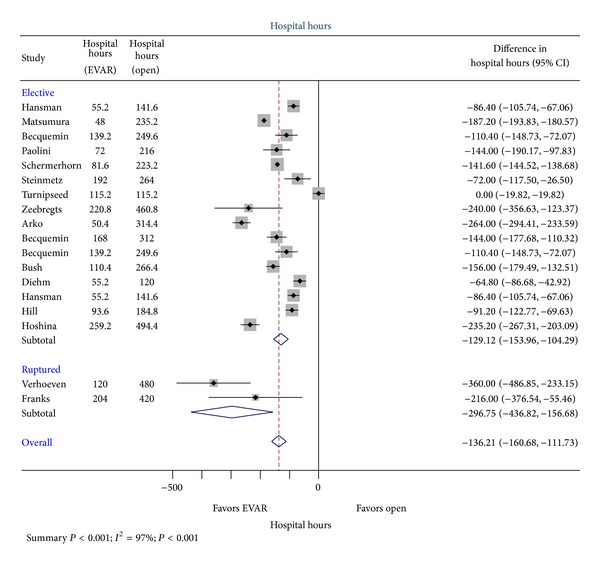
Comparison of reported hospital length of stay between OR and EVAR.

Sixteen trials were analyzed for the effect of surgical approach on hospital length of stay [6, 19, 20, 22, 28, 30, 32, 34, 36, 47–49]. An average decrease in hospital length of stay of 129.12 hrs (95% CI 104.29–153.96 hrs, *P* < 0.001) was observed in the EVAR group undergoing elective AAA repair (Figure 5). Two trials addressing ruptured AAA repair and hospital length of stay were analyzed [18, 50]. The average decrease in length of stay was 296.75 hrs (95% CI 156.68–436.82 hrs; *P* < 0.001) in the EVAR population when compared to open repair. Combined analysis of elective and open AAA repair with regard to hospital length of stay demonstrated a decrease in 136.21 hrs (95% CI 111.73–160.68 hrs; *I* ^2^ = 97%; *P* < 0.001) in favor of an endovascular approach.

## 4. Discussion

In this study of pooled population of 37,781 patients with known AAA who underwent either EVAR or OAR in both the elective as well as the ruptured setting, EVAR appears to be favored with lower rates of not only 30-day mortality and average hospital length of stay but also myocardial infarction that is potentially associated with significant cost reduction without compromising outcomes.

In a similar meta-analysis performed by Lovegrove et al. of 21,178 patients who underwent either EVAR or OAR for elective AAA repair, EVAR was associated with shorter intensive care unit, total hospital stay, less cardiac and respiratory complications, and lower mortality rates [51]. However, myocardial infarction was not evaluated and was included in the large cardiac complication definition. In the ruptured setting, EVAR was associated with lower mortality rates than OAR with no difference in cardiac complications [46]. In our pooled data of 37,781 patients, EVAR was favored over OAR with statistically significantly lower rates of mortality and myocardial infarction with associated shorter hospital length of stay in both elective and ruptured setting. This result was observed in both observational as well as randomized clinical trials (RCT) but with less heterogeneity noted with RCT that we postulate is most likely related to variable experience of operators in different sites in EVAR versus OAR. This is in contrast to large volume RCT where operators underwent significant training period prior to site initiation, potentially leading to less complication and less heterogeneity in outcomes.

Pooled analysis of both elective and ruptured studies seems to favor EVAR with regards to perioperative risk for MI with a trend towards statistical significance. This data should be interpreted in the context of inherit selection bias regarding the patient population referred for EVAR (typically infrarenal anatomy, accommodating neck size, lack of complex anatomy, typically asymptomatic, smaller diameter, and without significant anatomic variation) compared with open repair. Additionally, patients referred for EVAR often have more significant comorbidity or acute illness precluding an open repair. Thus, this is critical in a population where the incidence of cardiovascular events is high at baseline to include postoperative MI in both the elective and the ruptured setting to identify an intervention that is associated over all with lower rates of postoperative MI.

There are limitations to this study design to include the observational design, heterogeneity of trials analyzed, variability in reporting various patient outcomes, and lack of individual patient data for covariates.

This study is one of the largest pooled data analysis of patients with known AAA who underwent either EVAR or OAR in the elective as well as the emergent (ruptured) setting. We demonstrated a statistically significant reduction in odds of 30-day mortality, myocardial infarction, and average hospital length of stay in AAA patients undergoing EVAR regardless of whether the procedure was elective or emergent in a large pooled patient's sample.

## 5. Conclusions

EVAR has lower rates of 30-day mortality, 30-day myocardial infarction, and associated hospital length of stay based on our analysis of the pooled data from both observation and randomized control trials involving endovascular and open approach to elective and ruptured AAA repair.

## Figures and Tables

**Table 1:** Baseline article demographics separated based on trial design and elective versus ruptured repair.

Randomized control trials	Publication year	EVAR/OAR, *n*	Age (EVAR/OAR)	AAA size, cm (EVAR/OAR)
Matsumura et al. [19]	2003	235/99	73 ± 0.5/70.1 ± 0.8	5.6 ± 0.1/5.9 ± 0.1
Becquemin et al. [20]	2011	150/149	68.9 ± 7.7/70 ± 7.1	5.5 ± 0.5/5.6 ± 0.7
Prinssen et al. [21]	2004	171/174	70.7 ± 6.6/69.5 ± 6.8	6.1 ± 0.9/6.0 ± 0.9
Greenhalgh et al. [2]	2004	626/626	74.1 ± 6.1/74 ± 6.1	6.4 ± 0.9/6.5 ± 1.0

Observational trials				
Elective repairs				
Hansman et al. [22]	2003	50/50	72.5 ± 8.4/72.1 ± 6.3	5.5 ± 0.9/6.2 ± 1.3
Hynes and Sultan [52]	2007	62/52	72.6 ± 6.3/74.6 ± 7.3	5.4 ± 1.1/6.2 ± 1.6
Mistry et al. [53]	2007	122/156	66/66	5.7 ± 1.0/5.7 ± 1.0
Moore et al. [23]	1999	100/100	74.7 ± 7.9/72.9 ± 7.9	5.6 ± 1.1/5.9 ± 1.2
May et al. [24]	1998	108/195	70/69	5.3/5.6
May et al. [25]	2001	148/135	72/69	NR
Moore et al. [54]	2003	573/111	72.8 ± 7.8/71.6 ± 7.0	NR
Paolini et al. [47]	2008	81/69	83.7 ± 3.2/83.2 ± 2.8	5.8 ± 1.0/6.2 ± 1.3
Porcellini et al. [26]	2007	11/14	71.3/69.6	5.9/6.8
Raval and Eskandari [27]	2012	1634/391	>80/>80	NR
Schermerhorn et al. [28]	2008	22830/22830	76/76	NR
Schouten et al. [29]	2005	55/69	74 ± 7.0/74 ± 6.0	6.0/6.0
Steinmetz et al. [30]	2010	148/134	78/76	5.7 ± 0.9/5.7 ± 1.1
Tefera et al. [31]	2004	61/23	74/74	6.0/6.0
Teufelsbauer et al. [55]	2002	206/248	73.4/70.6	NR
Turnipseed et al. [32]	2003	70/96	73/70	5.9/5.8
Wahlgren and Malmstedt [56]	2008	217/483	74 ± 7/71 ± 8	NR
Wang and Carpenter [57]	2008	2664/334	73.1 ± 7.8/70 ± 7.8	5.6 ± 1.0/5.7 ± 1.2
Zeebregts et al. [48]	2004	93/82	70.9 ± 8.8/69.1 ± 7.7	6.0 ± 1.1/6.4 ± 1.3
Aarts et al. [33]	2005	99/116	NR	5.8/6.0
Arko et al. [34]	2002	174/243	73.5 ± 8.1/73.4 ± 7.8	5.8 ± 0.9/6.4 ± 0.2
de Bruin et al. [35]	2010	173/178	70.7 ± 6.6/69.6 ± 6.8	NR
Becquemin et al. [36]	2000	73/107	70/69	5.0 ± 0.5/5.1 ± 1.1
Brown et al. [58]	2011	543/539	74.2 ± 6/74 ± 6.1	6.5 ± 0.9/6.5 ± 0.9
Bush et al. [49]	2007	788/1580	72.9 ± 6.7/71.8 ± 6.4	NR
Cao et al. [37]	2004	534/585	73/72	5.2/5.6
de Virgilio et al. [59]	1999	83/63	73/68	NR
Chan et al. [38]	2007	157/329	75/72	6.1/6.3
Chisci et al. [39]	2009	74/61	77.5 ± 7/67.8 ± 8.7	6.2/6.3
Criado et al. [40]	2003	240/126	75.5/70	5.70.96/NR
Elkouri et al. [41]	2004	94/261	77/73	5.7/5.7
García-Madrid et al. [42]	2004	53/30	73/70	6.2/6.4
Gawenda et al. [43]	2003	10/16	57/52.5	NR
Gou$\ddot{\text{e}}$ffic et al. [60]	2005	209/289	71 ± 8/69 ± 8	5.2 ± 0.9/5.4 ± 1.5
Gupta et al. [44]	2012	369/282	56/56	NR
Hill et al. [61]	2002	79/70	74 ± 8/72 ± 8	5.9 ± 0.9/5.9 ± 1.4
Diehm et al. [62]	2008	25/25	62 ± 2.8/59 ± 3.9	5.0 ± 0.7/5.5 ± 1.0
Hoshina et al. [45]	2012	326/247	75.8 ± 6.3/74.7 ± 8	5.2 ± 1.0/5.4 ± 1.1

Ruptured Repairs				
Mcphee et al. [7]	2009	3179/24571	74.3/73	N/A
Nedeau et al. [8]	2012	19/55	78.2/76.3	N/A
Saqib et al. [9]	2012	37/111	74.9 ± 8.2/75.6 ± 8.4	N/A
Sarac et al. [10]	2011	32/128	80.5/72	N/A
Sharif et al. [11]	2007	52/74	74/74	N/A
Vaddineni et al. [12]	2005	9/15	70.8 ± 2.9/72.2 ± 5.5	N/A
Verhoeven et al. [13]	2008	36/89	72 ± 8.7/72 ± 8.7	N/A
Visser et al. [14]	2009	58/143	73.2 ± 8.6/73.5 ± 7.5	N/A
Alsac et al. [15]	2005	17/20	72.9 ± 9.8/72.8 ± 7.8	N/A
Coppi et al. [16]	2006	25/33	72.2 ± 8.2/74.3 ± 7.1	N/A
Bosch et al. [17]	2010	33/91	81/77	N/A
Franks et al. [18]	2006	21/22	73.7 ± 6.3/71.8 ± 5.7	N/A

## References

[1] Lederle FA, Freischlag JA, Kyriakides TC (2009). Outcomes following endovascular vs open repair of abdominal aortic aneurysm: a randomized trial. *The Journal of the American Medical Association*.

[2] Greenhalgh RM, Brown LC, Kwong GPS, Thompson SG (2004). Comparison of endovascular aneurysm repair with open repair in patients with abdominal aortic aneurysm (EVAR trial 1), 30-day operative mortality results: randomized controlled trial. *The Lancet*.

[3] Greenhalgh RM, Brown LC, Powell JT (2010). Endovascular versus open repair of abdominal aortic aneurysm. *The New England Journal of Medicine*.

[4] Blankensteijn JD, de Jong SECA, Prinssen M (2005). Two-year outcomes after conventional or endovascular repair of abdominal aortic aneurysms. *The New England Journal of Medicine*.

[5] Stroup DF, Berlin JA, Morton SC (2000). Meta-analysis of observational studies in epidemiology: a proposal for reporting. *The Journal of the American Medical Association*.

[6] http://www.ohri.ca/programs/clinical_epidemiology/oxford.htm.

[7] McPhee J, Eslami MH, Arous EJ, Messina LM, Schanzer A (2009). Endovascular treatment of ruptured abdominal aortic aneurysms in the United States (2001–2006): a significant survival benefit over open repair is independently associated with increased institutional volume. *Journal of Vascular Surgery*.

[8] Nedeau AE, Pomposelli FB, Hamdan AD (2012). Endovascular versus open repair for ruptured abdominal aortic aneurysm. *Journal of Vascular Surgery*.

[9] Saqib N, Park SC, Park T (2012). Endovascular repair of ruptured abdominal aortic aneurysm does not confer survival benefits over open repair. *Journal of Vascular Surgery*.

[10] Sarac TP, Bannazadeh M, Rowan AF (2011). Comparative predictors of mortality for endovascular and open repair of ruptured infrarenal abdominal aortic aneurysms. *Annals of Vascular Surgery*.

[11] Sharif MA, Lee B, Makar RR, Loan W, Soong CV (2007). Role of the Hardman index in predicting mortality for open and endovascular repair of ruptured abdominal aortic aneurysm. *Journal of Endovascular Therapy*.

[12] Vaddineni SK, Russo GC, Patterson MA, Taylor SM, Jordan WD (2005). Ruptured abdominal aortic aneurysm: a retrospective assessment of open versus endovascular repair. *Annals of Vascular Surgery*.

[13] Verhoeven EL, Kapma MR, Groen H (2008). Mortality of ruptured abdominal aortic aneurysm treated with open or endovascular repair. *Journal of Vascular Surgery*.

[14] Visser JJ, Williams M, Kievit J, Bosch JL (2009). Prediction of 30-day mortality after endovascular repair or open surgery in patients with ruptured abdominal aortic aneurysms. *Journal of Vascular Surgery*.

[15] Alsac J-M, Desgranges P, Kobeiter H, Becquemin J-P (2005). Emergency endovascular repair for ruptured abdominal aortic aneurysms: feasibility and comparison of early results with conventional open repair. *European Journal of Vascular and Endovascular Surgery*.

[16] Coppi G, Silingardi R, Gennai S, Saitta G, Ciardullo AV (2006). A single-center experience in open and endovascular treatment of hemodynamically unstable and stable patients with ruptured abdominal aortic aneurysms. *Journal of Vascular Surgery*.

[17] Bosch JAT, Teijink JAW, Willigendael EM, Prins MH (2010). Endovascular aneurysm repair is superior to open surgery for ruptured abdominal aortic aneurysms in EVAR-suitable patients. *Journal of Vascular Surgery*.

[18] Franks S, Lloyd G, Fishwick G, Bown M, Sayers R (2006). Endovascular treatment of ruptured and symptomatic abdominal aortic aneurysms. *European Journal of Vascular and Endovascular Surgery*.

[19] Matsumura JS, Brewster DC, Makaroun MS, Naftel DC (2003). A multicenter controlled clinical trial of open versus endovascular treatment of abdominal aortic aneurysm. *Journal of Vascular Surgery*.

[20] Becquemin J-P, Pillet J-C, Lescalie F (2011). A randomized controlled trial of endovascular aneurysm repair versus open surgery for abdominal aortic aneurysms in low- to moderate-risk patients. *Journal of Vascular Surgery*.

[21] Prinssen M, Verhoeven ELG, Buth J (2004). A randomized trial comparing conventional and endovascular repair of abdominal aortic aneurysms. *The New England Journal of Medicine*.

[22] Hansman MF, Neuzil D, Quigley TM (2003). A comparison of 50 initial endoluminal endograft repairs for abdominal aortic aneurysm with 50 concurrent open repairs. *The American Journal of Surgery*.

[23] Moore WS, Kashyap VS, Vescera CL, Quiñones-Baldrich WJ (1999). Abdominal aortic aneurysm: a 6-year comparison of endovascular versus transabdominal repair. *Annals of Surgery*.

[24] May J, White GH, Yu W (1998). Concurrent comparison of endoluminal versus open repair in the treatment of abdominal aortic aneurysms: analysis of 303 patients by life table method. *Journal of Vascular Surgery*.

[25] May J, White GH, Waugh R (2001). Improved survival after endoluminal repair with second-generation prostheses compared with open repair in the treatment of abdominal aortic aneurysms: a 5-year concurrent comparison using life table method. *Journal of Vascular Surgery*.

[26] Porcellini M, Nastro P, Bracale U, Brearley S, Giordano P (2007). Endovascular versus open surgical repair of abdominal aortic aneurysm with concomitant malignancy. *Journal of Vascular Surgery*.

[27] Raval MV, Eskandari MK (2012). Outcomes of elective abdominal aortic aneurysm repair among the elderly: endovascular versus open repair. *Surgery*.

[28] Schermerhorn ML, O’Malley AJ, Jhaveri A, Cotterill P, Pomposelli F, Landon BE (2008). Endovascular vs. open repair of abdominal aortic aneurysms in the medicare population. *The New England Journal of Medicine*.

[29] Schouten O, van Waning VH, Kertai MD (2005). Perioperative and long-term cardiovascular outcomes in patients undergoing endovascular treatment compared with open vascular surgery for abdominal aortic aneurysm or Iliaco-Femoro-Popliteal bypass. *American Journal of Cardiology*.

[30] Steinmetz E, Abello N, Kretz B, Gauthier E, Bouchot O, Brenot R (2010). Analysis of outcome after using high-risk criteria selection to surgery versus endovascular repair in the modern era of abdominal aortic aneurysm treatment. *European Journal of Vascular and Endovascular Surgery*.

[31] Tefera G, Carr SC, Turnipseed WD (2004). Endovascular aortic repair or minimal incision aortic surgery: which procedure to choose for treatment of high-risk aneurysms?. *Surgery*.

[32] Turnipseed W, Tefera G, Carr S (2003). Comparison of minimal incision aortic surgery with endovascular aortic repair. *The American Journal of Surgery*.

[33] Aarts F, van Sterkenburg S, Blankensteijn JD (2005). Endovascular aneurysm repair versus open aneurysm repair: comparison of treatment outcome and procedure-related reintervention rate. *Annals of Vascular Surgery*.

[34] Arko FR, Lee WA, Hill BB (2002). Aneurysm-related death: primary endpoint analysis for comparison of open and endovascular repair. *Journal of Vascular Surgery*.

[35] de Bruin JL, Baas AF, Buth J (2010). Long-term outcome of open or endovascular repair of abdominal aortic aneurysm. *The New England Journal of Medicine*.

[36] Becquemin J-P, Bourriez A, D’Audiffret A (2000). Mid-term results of endovascular versus open repair for abdominal aortic aneurysm in patients anatomically suitable for endovascular repair. *European Journal of Vascular and Endovascular Surgery*.

[37] Cao P, Verzini F, Parlani G (2004). Clinical effect of abdominal aortic aneurysm endografting: 7-year concurrent comparison with open repair. *Journal of Vascular Surgery*.

[38] Chan YC, Morales JP, Gulamhuseinwala N (2007). Large infra-renal abdominal aortic aneurysms: endovascular versus open repair—single centre experience. *International Journal of Clinical Practice*.

[39] Chisci E, Kristmundsson T, de Donato G (2009). The AAA with a challenging neck: outcome of open versus endovascular repair with standard and fenestrated stent-grafts. *Journal of Endovascular Therapy*.

[40] Criado FJ, Fairman RM, Becker GJ (2003). Talent LPS AAA stent graft: results of a pivotal clinical trial. *Journal of Vascular Surgery*.

[41] Elkouri S, Gloviczki P, McKusick MA (2004). Perioperative complications and early outcome after endovascular and open surgical repair of abdominal aortic aneurysms. *Journal of Vascular Surgery*.

[42] García-Madrid C, Josa M, Riambau V, Mestres C, Muntaña J, Mulet J (2004). Endovascular versus open surgical repair of abdominal aortic aneurysm: a comparison of early and intermediate results in patients suitable for both techniques. *European Journal of Vascular and Endovascular Surgery*.

[43] Gawenda M, Zaehringer M, Brunkwall J (2003). Open versus endovascular repair of para-anastomotic aneurysms in patients who were morphological candidates for endovascular treatment. *Journal of Endovascular Therapy*.

[44] Gupta PK, Ramanan B, Lynch TG (2012). Endovascular repair of abdominal aortic aneurysm does not improve early survival versus open repair in patients younger than 60 years. *European Journal of Vascular and Endovascular Surgery*.

[45] Hoshina K, Hosaka A, Takayama T (2012). Outcomes after open surgery and endovascular aneurysm repair for abdominal aortic aneurysm in patients with massive neck atheroma. *European Journal of Vascular and Endovascular Surgery*.

[46] Hinchliffe RJ, Bruijstens L, MacSweeney STR, Braithwaite BD (2006). A randomised trial of endovascular and open surgery for ruptured abdominal aortic aneurysm—results of a pilot study and lessons learned for future studies. *European Journal of Vascular and Endovascular Surgery*.

[47] Paolini D, Chahwan S, Wojnarowski D, Pigott JP, LaPorte F, Comerota AJ (2008). Elective endovascular and open repair of abdominal aortic aneurysms in octogenarians. *Journal of Vascular Surgery*.

[48] Zeebregts CJ, Geelkerken RH, van der Palen J, Huisman AB, de Smit P, van Det RJ (2004). Outcome of abdominal aortic aneurysm repair in the era of endovascular treatment. *The British Journal of Surgery*.

[49] Bush RL, Johnson ML, Hedayati N, Henderson WG, Lin PH, Lumsden AB (2007). Performance of endovascular aortic aneurysm repair in high-risk patients: results from the veterans affairs national surgical quality improvement program. *Journal of Vascular Surgery*.

[50] Verhoeven EL, Kapma MR, Groen H (2008). Mortality of ruptured abdominal aortic aneurysm treated with open or endovascular repair. *Journal of Vascular Surgery*.

[51] Lovegrove RE, Javid M, Magee TR, Galland RB (2008). A meta-analysis of 21178 patients undergoing open or endovascular repair of abdominal aortic aneurysm. *The British Journal of Surgery*.

[52] Hynes N, Sultan S (2007). A prospective clinical, economic, and quality-of-life analysis comparing endovascular aneurysm repair (EVAR), open repair, and best medical treatment in high-risk patients with abdominal aortic aneurysms suitable for EVAR: the Irish patient trial. *Journal of Endovascular Therapy*.

[53] Mistry PP, Becker P, van Marle J (2007). A prospective comparison of secondary interventions and mortality in open and endovascular infrarenal abdominal aortic aneurysm repair. *South African Journal of Surgery*.

[54] Moore WS, Matsumura JS, Makaroun MS (2003). Five-year interim comparison of the Guidant bifurcated endograft with open repair of abdominal aortic aneurysm. *Journal of Vascular Surgery*.

[55] Teufelsbauer H, Prusa AM, Wolff K (2002). Endovascular stent grafting versus open surgical operation in patients with infrarenal aortic aneurysms: a propensity score-adjusted analysis. *Circulation*.

[56] Wahlgren CM, Malmstedt J (2008). Outcomes of endovascular abdominal aortic aneurysm repair compared with open surgical repair in high-risk patients: results from the Swedish vascular registry. *Journal of Vascular Surgery*.

[57] Wang GJ, Carpenter JP (2008). The Powerlink system for endovascular abdominal aortic aneurysm repair: six-year results. *Journal of Vascular Surgery*.

[58] Brown LC, Thompson SG, Greenhalgh RM, Powell JT (2011). Incidence of cardiovascular events and death after open or endovascular repair of abdominal aortic aneurysm in the randomized EVAR trial 1. *The British Journal of Surgery*.

[59] de Virgilio C, Bui H, Donayre C (1999). Endovascular versus open abdominal aortic aneurysm repair: a comparison of cardiac morbidity and mortality. *Archives of Surgery*.

[60] Gouëffic Y, Becquemin J-P, Desgranges P, Kobeiter H (2005). Midterm survival after endovascular versus open repair of infrarenal aortic aneurysms. *Journal of Endovascular Therapy*.

[61] Hill BB, Wolf YG, Lee WA (2002). Open versus endovascular AAA repair in patients who are morphological candidates for endovascular treatment. *Journal of Endovascular Therapy*.

[62] Diehm N, Tsoukas AI, Katzen BT (2008). Matched-pair analysis of endovascular versus open surgical repair of abdominal aortic aneurysms in young patients at low risk. *Journal of Vascular and Interventional Radiology*.

